# A Knowledge-Fusion Ranking System with an Attention Network for Making Assignment Recommendations

**DOI:** 10.1155/2020/6748430

**Published:** 2020-12-23

**Authors:** Canghong Jin, Yuli Zhou, Shengyu Ying, Chi Zhang, Weisong Wang, Minghui Wu

**Affiliations:** ^1^Zhejiang University City College, Hangzhou, Zhejiang, China; ^2^Zhejiang University, Hangzhou, Zhejiang, China; ^3^Zhejiang Xuehai Education Technology Co., Ltd., Hangzhou, Zhejiang, China

## Abstract

In recent decades, more teachers are using question generators to provide students with online homework. Learning-to-rank (LTR) methods can partially rank questions to address the needs of individual students and reduce their study burden. Unfortunately, ranking questions for students is not trivial because of three main challenges: (1) discovering students' latent knowledge and cognitive level is difficult, (2) the content of quizzes can be totally different but the knowledge points of these quizzes may be inherently related, and (3) ranking models based on supervised, semisupervised, or reinforcement learning focus on the current assignment without considering past performance. In this work, we propose KFRank, a knowledge-fusion ranking model based on reinforcement learning, which considers both a student's assignment history and the relevance of quizzes with their knowledge points. First, we load students' assignment history, reorganize it using knowledge points, and calculate the effective features for ranking in terms of the relation between a student's knowledge cognitive and the question. Then, a similarity estimator is built to choose historical questions, and an attention neural network is used to calculate the attention value and update the current study state with knowledge fusion. Finally, a rank algorithm based on a Markov decision process is used to optimize the parameters. Extensive experiments were conducted on a real-life dataset spanning a year and we compared our model with the state-of-the-art ranking models (e.g., ListNET and LambdaMART) and reinforcement-learning methods (such as MDPRank). Based on top-*k* nDCG values, our model outperforms other methods for groups of average and weak students, whose study abilities are relatively poor and thus their behaviors are more difficult to predict.

## 1. Introduction

Educational data mining is an emerging discipline, concerned with developing methods for exploring the unique and increasingly large-scale data that come from educational settings and using those methods to understand students and the settings which they learn in better. In recent years, physical bricks and mortar classrooms are starting to lose their monopoly as a place of learning. The Internet has made online learning possible, and many researchers and educators are interested in online learning to enhance and improve students' learning outcomes while mitigating the reduction in resources [1]. Online learning platforms include Coursera, MOOC, and Udacity. Online assignments, such as quizzes, practice exercises, virtual labs, online literature searches, and simulations, play a critical role in online learning [2].

One of the most important tasks of an online assignment system in educational data mining is to find suitable questions for students according to their ability. An online assignment system can make learning more efficient. It can evaluate study performance and identify at-risk students, who can be given further help. For example, from a log of assignment results, we can identify topics that a student has poorly mastered and recommend to them related questions to improve their knowledge. Thus, the task is to rank a large number of questions and recommend only relevant questions to students.

An automated process for producing an online assignment works as follows:

- Several questions, which are organized into an assignment, are assigned by a teacher weekly following the syllabus. Students are expected to finish them on time.
- The system can check the answers automatically, calculate the students' marks, and generate a report for each assignment.
- Since the number of candidate questions is too large to finish in one assignment, the recommendation system should be able to choose suitable questions and discard those that are too easy or too difficult.

Note that the goal of an assignment recommendation task is not whether the question can be answered correctly; that is, it is not a classification problem. Rather, questions should be ranked according to the importance of the topic in improving the student's ability. The aim is to find the questions with the highest benefit for students.

In previous studies, this problem has been tackled by supervised and semisupervised ranking methods [3–5]. In particular, state-of-the-art reinforcement learning has been used, which considers the problem as a process of sequential decision-making and learns model parameters through maximizing the rewards accumulated from all decisions [6]. MDPRank is a ranking model based on a Markov decision process (MDP). It treats documents as states and ranks the position of documents at each iteration. However, the current MDPRank is imperfect if we apply it to our assignment recommendation system directly. It is obvious that a student's performance in an assignment not only depends on the questions, such as the marks for each question, the types of question, and the difficulty of each question, but also depends on their current knowledge, especially for those questions with similar knowledge points. Other researches [7, 8] are close to our recommend target but both of them are based on study cognition and semantic content of questions, which is more complex than our situations

**Figure 1:**
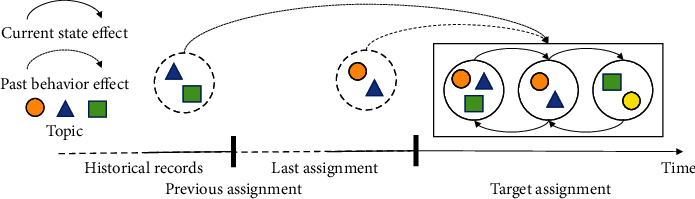
Motivation and intuition of our solution. For a student, the right-hand box is the target assignment and the dashed circles are previously completed assignments. The geometric forms inside each period are the knowledge points in each question.

In this article, we illustrate our motivation in Figure 1, which shows the relations between questions from different assignments. Our intuition is that the ranking is influenced by two dimensions: how questions have been answered in the same assignment and how questions were answered in previous assignments.

Based on the above intuition, we propose a knowledge-fusion ranking system using an attention network, KFRank, which can improve the reliability and accuracy of ranking using the relations between knowledge points. Compared with the state-of-the-art learning-to-rank (LTR) and reinforcement-learning methods, our KFRank method has the following advantages:

- KFRank considers the ranking problem as having multilevel dimensions and generates effective cognitive features for learning models.
- KFRank utilizes knowledge points and integrates them during training. We build a cluster of questions using the knowledge points and generate an attention network to pretrain the terms in questions. These are represented in vectors of questions in the classification phase.
- KFRank is based on an MDP but rebuilds the environment by considering multiple factors: questions in the current assignment and results from previous similar assignments.

The rest of the paper is organized as follows: first, we formulate the problem and introduce the concepts used in the assignment ranking problem in Section 2.  Section 3 gives the architecture of KFRank and proposes the attention network for training. Next, in Section 4, we describe our experiments with real-life datasets and compare the performance of the proposed model with other methods. Related work is discussed in Section 5, and Section 6 concludes the work.

## 2. Preliminaries

In this section, we first formally formulate the assignment ranking problem and then briefly introduce the LTR and reinforcement-learning methods for solving this problem.

### 2.1. Problem Definition

**Definition 1 .**

An assignment *ω*=(*Q*, *p*, *t*, *λ*), where *Q*={*q*_1_, *q*_2_,…, *q*_|*q*|_} is a set of questions, *p* is a unique student, *t* is the assignment time, and *λ* is the score for *q*.

**Definition 2 .**

The knowledge points of a question *𝒪* are a set of knowledge points *o*_*q*_, which belong to question *q*.

**Definition 3 .**

Our assignment ranking problem is to rank questions in an assignment by predicting the performance of each student based on the difficulty of the questions. Questions higher in the list are relatively easier for a particular student than other questions.

### 2.2. Ranking Using Supervised Learning

LTR is a sorting method based on supervised learning. The user-item scoring matrix is produced by a recommendation algorithm after learning from a training set. Here, different sorting techniques, such as pointwise [9], pairwise [10], and listwise [11], can be used to obtain a sorting model. In the test phase, the system generates an ordered list of items for target users using the trained model.

LTR can be online or offline. In offline approaches, the training set is produced by human assessors, which is time-consuming and expensive. In contrast, an online LTR system collects data when users interact with the system, such as by clicking, moving a mouse, and entering a query string.

### 2.3. Ranking Using Reinforcement Learning

Reinforcement learning is a branch of artificial intelligence. It is good at controlling an agent who can act autonomously in a specific environment and continuously improve their behavior. Suitable problems for reinforcement learning involve learning how to do tasks and how to map the environment into actions that maximize the rewards. In reinforcement learning, the learner is a decision-making agent who is not told what to do. Instead, they attempt a task repeatedly to find the behavior that gives the greatest reward. By giving each question a reward, reinforcement learning can learn how to rank them in an assignment as a closed-loop control problem.

## 3. The Kfrank Model

Three aspects of the KFRank model are presented in this section: (1) an MDP for ranking, (2) a knowledge-fusion model for updating the environment, and (3) the architecture and algorithm (Algorithm 1) of KFRank.

### 3.1. Ranking Using an MDP

Analyzing study performance can be formalized as an MDP, in which the construction of a list of ranked questions can be considered as a sequential decision-making process in which each time step corresponds to selecting a question for a corresponding position. We propose a tuple 〈*S*, *A*, *T*, ℛ, *π*, ℒ, *χ*〉 to illustrate KFRank by states, actions, transition, reward, policy, history, and rebuilder, which are defined as follows.

#### 3.1.1. States

*S* is a set of states that represent the environment of the current assignments. In ranking, the agent should know the current positions as well as the remaining questions. Thus, state *S*_*t*_ for step *t* is [*t*, *X*_*t*_], where in our model *X* is initially treated as a given assignment *ω*, and *X*_*t*_ are the questions still to be ranked *Q*_*t*_.

#### 3.1.2. Actions

*A* are a discrete set of actions that an agent can take in which available actions can depend on the state *S*, denoted as *A*(*s*_*t*_). At step *t*, *a*_*t*_ ∈ *A*(*s*_*t*_) is used to calculate the value of each *q* in *ω* and to select a question *q*_*m*_(*a*_*t*_) for the ranking position *t*+1, where *m*(*a*_*t*_) is the index of the question selected by action *a*_*t*_.

#### 3.1.3. Transition

*T*(*S*, *A*) is a function that maps state *S*_*t*_ and action *A*_*t*_ to a new state *S*_*t*+1_ as *S* × *A*⟶*S*. At step *t*, action *a*_*t*_ selects *q*_*m*_(*a*_*t*_) and removes it from *Q*_*t*_ as follows:

(1) $$\begin{array}{c} s_{t+1}=T\left(s_{t},a_{t}\right)=\left[t+1,Q_{t}\backslash q_{m}\left(a_{t}\right)\right]. \end{array}$$

#### 3.1.4. Reward

The state value function *V* : *S*⟶ℛ is a scalar evaluation, estimating the quality of the entire list of ranked questions (an assignment) based on the input state *S*. Here, we define the value function as DCG:

(2) $$\begin{array}{c} {ℛ}_{\text{DCG}\left(s_{t},a_{t}\right)}=\left\{\begin{array}{cc} 2^{y_{m}\left(a_{t}\right)}-1, & \text{if }t=0,\\ \frac{2^{y_{m}\left(a_{t}\right)}-1}{\log_{2}\left(t+1\right)}, & \text{if }t=1, \end{array}\right. \end{array}$$

where *y*_*m*_(*a*_*t*_) is a relevance label for the current selected question *q*_*m*_(*a*_*t*_). In our model, we calculate *y*_*m*_(*a*_*t*_) according to a student's performance and the difficulty of a question. The difficulty *θ* of *q* is defined as

(3) $$\begin{array}{c} q\left(\theta \right)=\frac{\sum_{k=1}^{N}q\left(r=0\right)}{\sum_{k=1}^{N}q\left(r=1\right)+\sum_{k=1}^{N}q\left(r=0\right)}, \end{array}$$

where *r*=0 means the result is wrong and *r*=1 means the result is right. Thus, *y*_*m*_(*a*_*t*_) is defined as

(4) $$\begin{array}{c} y_{m}\left(a_{t}\right)=V\left(q\left(r\right)\right)\times q\left(\theta \right). \end{array}$$

#### 3.1.5. Policy

*π* is a function that takes a state as input and outputs a distribution over all possible actions *a* ∈ *A*(*s*). KFRank calculates the probability of selecting each question based on its current rank:

(5) $$\begin{array}{c} \pi \left(a_{t}|s_{t};w\right)=\frac{\exp\left\{w^{T}q_{m}\left(a_{t}\right)\right\}}{\sum_{a\in A\left(s_{t}\right)}\exp\left\{w^{T}q_{m}\left(a_{t}\right)\right\}}, \end{array}$$

where *w* ∈ *ℝ*^*K*^ are the model parameters, whose dimension is the same as that of the ranking feature.

In our case, the policy is an agent's strategy to rank the assignment by predicting the study results and measure the rewards by nDCG. Obviously, some policies are better than others, and there are multiple ways to assess them.

#### 3.1.6. History

ℒ is a set of historical assignment results for a group of students. Here, ℒ={*l*_*p*_^*q*^}, where *l* is a previous result, *q* is a question, and *p* is a student.

ℒ is designed as an information retrieval system and contains student IDs, questions, knowledge points, results, and operating time. Several metrics related to information retrieval can be used to compare the similarity of given questions and archived questions as follows:

(6) $$\begin{array}{c} D\left(ℒ,q,ℳ,k\right)=ℳ\left(o_{q_{i}\in ℒ},o_{q},k\right), \end{array}$$

where *q* is a question in *ω*, ℳ is an information retrieval search function, and *k* is the number of output results.

#### 3.1.7. Rebuilder

*χ* is a module that updates current states *s* to ${\hat{s}}_{t}$ with *𝒟* as input. We extract a student's performance on special knowledge points *o* from ℒ and predict their current ability using a recurrent neural network.

### 3.2. Basic Study State

We calculate the effective features for ranking in terms of the relation between a student's knowledge cognitive and the question. In this article, we first estimate the difficulty of each question using a correctness ratio. Then, for each student, their knowledge cognitive is measured as their average score for all completed assignments based on knowledge points. Here, a student's knowledge cognitive is dynamic and updated during the study process. Thus, if a student does very well on an assignment, all the related cognitive levels increase rapidly. On the other hand, the difficulty of a question is fixed or not easy to change, because it depends on the performance of all students.

Based on the student's knowledge cognitive and the difficulty of the questions, we utilize several similarity functions to construct the representation of the student's states for ranking according to the traditional learning-to-rank method, including Euclidean distance, Pearson's similarity, Manhattan distance, cosine similarity, and so on.

**Figure 2:**
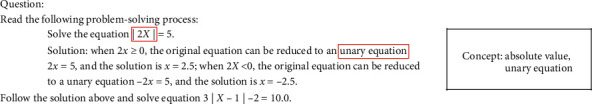
Typical question. In this question, there are two separate knowledge points: an unary equation and an absolute value. If a student answers the question right, both cognitive levels would be increased.

**Figure 3:**
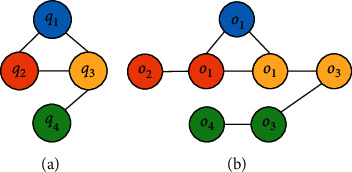
Relations between questions and knowledge points. (a) Each circle represents a question. (b) The correlations between questions. Knowledge points with the same color are from the same question; for example, *o*_1_ and *o*_2_ are two different knowledge points in *q*_2_, whereas *q*_1_ connects to *q*_2_ with knowledge point *o*_1_.

As shown in Figures 2 and 3, one question can contain several knowledge points and one knowledge point may have many related questions. Thus, for a given question with several knowledge points, we first calculate the state for each knowledge point and then merge all the related states. According to the relationship of knowledge points in the question, we can obtain many basic triples in the knowledge graph. We will introduce how to build a reasonable representation of knowledge points in Section 3.3.

### 3.3. Knowledge Representation with Trans*R*

Since the content of math questions can vary considerably, we use knowledge points to illustrate the relations between questions, as Figure 3 shows. From the knowledge points and the relations between them, which are manually marked, we can construct the triples in the knowledge graphs. In this project, the relations between knowledge points are classed as *contains*, *belongs*, and *equals*. The following is an example.

In triangle *A*, angle *B* is equal to 90° and angle *B* is a right angle.

Here, *triangle* is a knowledge point, the relation between *A* and *B* is *contains*, the relation between *B* and *A* is *belongs*, and the relation between *right angle* and 90° is *equals*. A triple is defined as (*h*, *r*, *t*), where *h*, *t* represent the embedding of knowledge points and *r* is the set of relations. From the knowledge graph, we can obtain the vectors of knowledge points using Trans*R* [12].

In Trans*R*, the score function is defined as

(7) $$\begin{array}{c} f_{r}\left(h,t\right)={\left\|h_{r}+r-t_{r}\right\|}_{2}^{2}, \end{array}$$

and the model convergence is based on minimizing

(8) $$\begin{array}{c} L=\sum_{\left(h,r,t\right)\in S}\sum_{\left(h^{\prime },r,t^{\prime }\right)\in S^{\prime }}\max\left(0,f_{r}\left(h,t\right)+\gamma -f_{r}\left(h^{\prime },t^{\prime }\right)\right), \end{array}$$

where *γ* is the margin, *S* is the set of correct triples, and *S*′ is the set of incorrect triples.

### 3.4. Attention-Based Knowledge-Fusion Model

**Figure 4:**
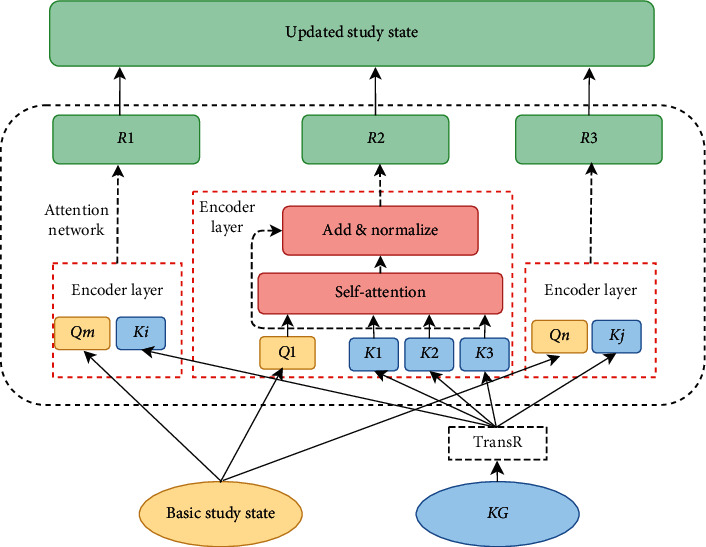
Updating state using knowledge fusion. *Q* is basic study state, *K* is the representation of knowledge points, which is calculated from knowledge graph with Trans*R*. *R* is the representation of output status.

To tackle the various knowledge points, we design an attention-based model using knowledge fusion. As Figure 4 shows, *Q* is the representation of a student's current knowledge, as noted in Section 3.2, and *k* is the embedding of concepts trained from Trans*R*. In the triple (query, key, value) in the attention mechanism, we set *Q* as *k*, the knowledge point as *q*, and the reward corresponding to the question as *v*. The model is trained in the same way as the encoder part in the transformer (Figure 4), the vectors of multiple knowledge points were incorporated into the basic study state with the way of attention. The output is the latest performance. It is equivalent to integrating the information of different knowledge points into the original state and getting a new vector to represent the current state. *R* in the figure represent the student's current knowledge state for each question. The status update process is shown in Algorithm 2.

**Figure 5:**
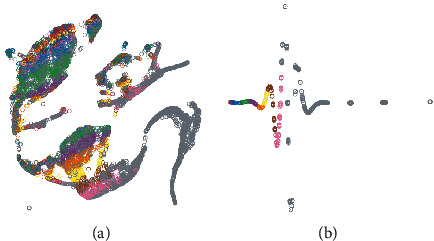
Visualization of state vectors. (a) Before attention model. (b) After attention model.

We show the study state vectors before and after the attention model in Figure 5. We select ten different knowledge points and related study states and then use the attention model to pretrain input vectors. Then, we use t-SNE to show the latent space representations of two states. Note that the study state after attention training is simple and is closely surrounded by knowledge points with the same color.

### 3.5. Overview of KFRank

**Figure 6:**
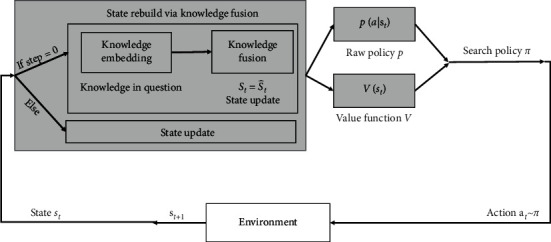
The agent-environment interaction in KFRank.

**Figure 7:**
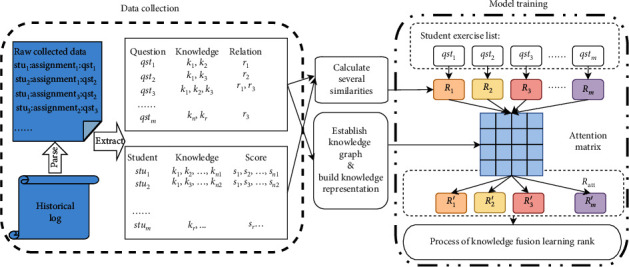
There are four steps in KFRank, from left to right: (1) data collection, knowledge tagging, and organization; (2) generating knowledge representations; (3) updating the current study performance environment with knowledge fusion; and (4) reinforcement-learning ranking.

Figures 6 and 7 illustrate the construction of the question ranking. For each question in an assignment, first, we extract related performance records. In each episode, the environment is updated with the current status from the knowledge-fusion model. Based on the policy and value function, the agent chooses the optimal action that gives the greatest long-term return. After taking action, the environment is updated.

The sorting construct for a given training document can be formalized as follows. A student's assignment is a query *ω*, which is a set of questions *Q* with length *M*. The initial state is *s*_0_=[0, *Q*]. At each step *t*=0,…, *M* − 1, the agent chooses the optimal action *a*_*t*_ to select *q*_*m*_(*q*_*t*_) from the set of questions *Q* as the rank *t* (lines 7 and 8 in Algorithm 1). The action is removed from *Q*_*t*_, as in equation (1) (lines 9 and 10 in Algorithm 1). We calculate *y*_*m*_(*a*_*t*_) using equation (3) and calculate the long-term return reward. The process is repeated until all of the *M* questions have been selected.

We propose to learn the parameters *w* in KFRank using a policy-gradient algorithm based on reinforcement learning [13]. The goal of this algorithm is to maximize the long-term return *G*_*t*_:

(9) $$\begin{array}{c} G_{t}=\sum_{k=1}^{M-t}\gamma^{k-1}r_{t+k}. \end{array}$$

In the algorithm, the gradient Δ_*w*_*J*(*w*) is calculated as

(10) $$\begin{array}{c} \Delta_{w}J\left(w\right)=\gamma^{t}G_{t}\nabla_{w}\log\;\pi \left(a_{t}|s_{t};w\right). \end{array}$$

At each iteration, an episode is sampled with the current policy. At each step *t*, the parameters *w* are adjusted according to ∇_*w*_log *π*(*a*_*t*_*|s*_*t*_; *w*), which maximizes the increase in the probability of repeating the action *a*_*t*_ for state *s*_*t*_. In this way, *G*_*t*_ moves the parameters in the direction that gives the greatest return for the action.

## 4. Performance Evaluation

### 4.1. Datasets

We conducted experiments to validate the performance of our method using a real-life dataset (Table 1), from two applications in pad: teacher client and student client. The data spanned the period from September 2017 to June 2018. There were nearly 70 million records for 40,000 students. Based on the student's historical activity and correctness rate, we selected a record of 300 students with relatively high quality of records. Then, we apportion the data into training and test sets, with a 70-30 split. After that, excellent teachers create the mathematics knowledge graph of middle school, which contains more than 700 nodes and 2000 relationship edges. Finally, we use Trans*R* approach to embed knowledge topic for learning.

**Figure 8:**
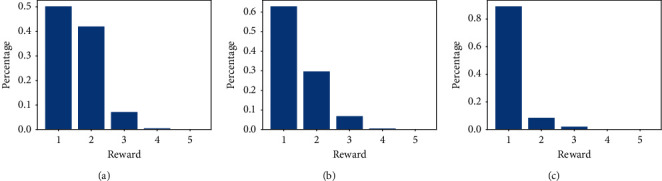
Distribution of rewards for three groups of students. (a) Merit student. (b) Average student. (c) Weak student.

To evaluate the effectiveness of the ranking, we split the students into three groups: *merit students*, *average students*, and *weak students*. In the dataset, almost 72 percent of the questions covered three or more knowledge points and less than 14 percent of the questions had only one knowledge point. The distributions of rewards for the three groups of students are shown in Figure 8.

### 4.2. Evaluation Criteria

We first calculate the score of each question for ranking by the performance result of students and difficulty of question. For example, for a given question, only 60 percent of students could choose the right answer, the difficulty of this question is 0.4. Then, we also give the correct and wrong answer with 5 and 1 score. Finally, students will get 2 points if they do the right questions, and only 0.4 if they make a mistake. For each assignment, according to the student's answer to each question, the rank score is calculated as the true value.

We use *n*DCG@*k* to measure the performance. To get *n*DCG@*k*, we first calculate DCG@*k*:

(11) $$\begin{array}{c} \text{DCG}\left(f\right)=\sum_{r=1}^{k}v_{r}\frac{1}{\log\left(1+r\right)}, \end{array}$$

where *r* is the rank of items in the recommendation list, *k* is the length of the recommendation list, *f* is the ranking function or algorithm, *v*_*r*_ is the value of the *r*th item, and 1/log(1+*r*) is the discount. *i*DCG is an ideal discounted cumulative gain, *i*DCG@*k* is also needed and calculated in a similar way. In *i*DCG@*k*, the questions in the recommendation list are ranked by their original values instead of by the ranking algorithm.

### 4.3. Experimental Results

Table 2 compares the performance of our model with other methods using *n*DCG@5 and *n*DCG@10. The higher the score, the better the performance. Of the LTR methods, CoordAscent, LambdaMART, and ListNet perform relatively well and better than the original reinforcement-learning method, MDPRank. KFRank with updated environments has the best nDCG value for nearly all groups of students.

From the results, it can be seen that the different methods have similar trends for performance for the three groups. For example, the nDCG scores for weak students are always higher than those for merit students, which means it is easier to make predictions for weak students. Students whose performance varies depending on the difficulty of the questions will have a better score.

**Figure 9:**
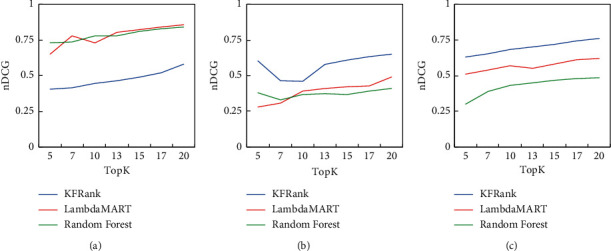
Comparison of LambdaMART, Random Forest, and KFRank. (a) Merit student. (b) Average student. (c) Weak student.

Then, we ran the experiment again and evaluated the stability of our method for various top *k* results. As shown in Figure 9, the performance of KFRank is generally better and more stable than Random Forest and LambdaMART in traditional learning-to-rank methods. From the results of KFRank, KFRank is superior in the performance of average and weak students, indicating that KFRank has greater help for students with poor knowledge.

## 5. Related Work

Related work can be classified into two categories, those based on LTR methods and those based on reinforcement learning.

### 5.1. Making Recommendations with LTR

Karatzoglou et al. [3] explained how the key ideas of different LTR methods can be applied to specific collaborative filtering methods. Sun et al. [4] applied the LTR method RankSVM to generate a list of recommended items for users. Yao et al. [5] applied the LTR method for item recommendation and integrated social information between users in the training of the Listwise model to improve the quality of a sorted list of items. Canuto et al. [22] applied the LTR method to learn automatically how to sort labels. They compared the performance of eight different methods of recommending labels. Ifada et al. [23] developed a novel LTR method Go-Rank for a label-based project recommendation system. They directly optimized the graded average precision, resulting in an optimized list of recommended items. Huang et al. [24] reviewed recent research into recommendation algorithms based on LTR. They generalized, compared, and analyzed problem definitions, key technologies, utility evaluations, and progress. Finally, they discussed and forecast the trends for recommendation algorithms based on LTR.

### 5.2. Making Recommendations with Advanced Reinforcement Learning

Shani et al. [25] proposed an MDP-based collaborative filtering model, which uses a finite window for history, instead of an unbounded one, to define the current state. It can be regarded as approximating a partial observable MDP (POMDP). Since POMDP has high computational and representational complexity, various strategies have been suggested for simplifying it, such as policy-based optimization [26], value function approximation [27], and stochastic sampling [28]. Regarding sequential decision problems, Sunehag et al. [29] designed a reinforcement-learning agent using high-dimensional combinatorial slate-action spaces and achieved remarkable results. As ranking is a key issue in practical recommendation problems, any improvements in ranking contribute significantly to reinforcement recommendation systems. Zhang et al. [30] used a log-based document reranking modeled as a POMDP. Wei et al. [6] proposed a novel LTR model based on a MDP, referred to as MDPRank, which directly optimizes a ranking using a MDP. FAIR-PG-Rank recommends items via a policy-gradient approach which could satisfy fairness of exposure constraints with respect to items [31]. A similar idea in the article is generating unified term impact (UTI) during the indexing time and combining into a hybrid model to improve the accuracy [32]. Since the relationship between study performance and exam results is much complicated, article [33] finds the correspondence of input values and predicts targets which is not a one-to-one relation, treats the classification task as a fuzzy geometrical problem, and proposes a fuzzy similarity approach to solve the problem [34, 35].

## 6. Conclusions

Assignment recommendation is an essential and trick task in online study research. In this paper, we investigated how to predict the performance of students by using assignment ranking mechanisms. Based on traditional learning-to-rank models, we proposed a knowledge-fusion model with an attention network named KFRank, which employs two novel features compared to previous methods: (1) an attention network for capturing multiple knowledge factors in human behavior and (2) a reinforcement-learning module for ranking questions by their predictable reward. Our model could capture both current study status and previous study performance of similar math topics. Extensive experiments on a real-world dataset with three different levels of students showed that KFRank significantly outperforms other methods in most cases.

In the future, there are still some directions for further studies. First, besides the historical log, we would like to measure the study performance in more aspects, for example, by studying cognitive model. Second, for the policy strategy, some network optimization [36] and fuzzy theories could be introduced in our model [34]. Finally, as our KFRank is a general framework, we will test its performance on other disciplines (e.g., click-through rate prediction) and, meanwhile, on the similar applications in other domains, such as the user behavior of customers in e-commerce.

## Figures and Tables

**Algorithm 1:**
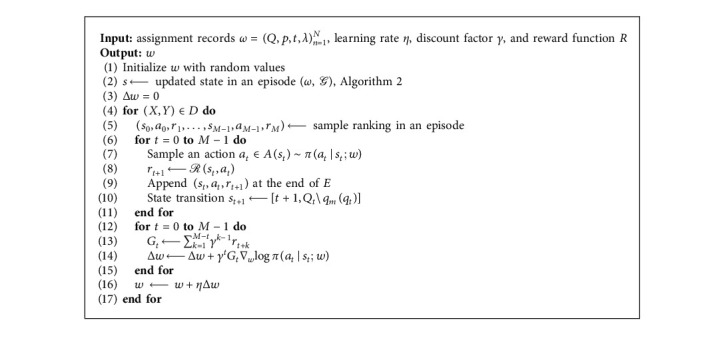
KFRank learning.

**Algorithm 2:**
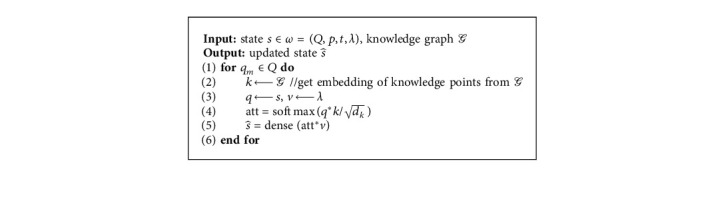
State updating in an episode.

**Table 1:** Summary of the dataset.

Metric	Merit	Average	Weak
Number of students	100	100	100
Number of quizzes	16,251	21,684	24,418
Number of knowledge points	354	440	401
Number of records	65,546	68,011	73,232
Ratio correct	0.92	0.61	0.21

**Table 2:** *n*DCG of ranking methods.

Method	Merit student		Average student		Weak student	
	@5	@10	@5	@10	@5	@10
MART [14]	0.701	0.751	0.309	0.319	0.272	0.384
RankNet [15]	0.507	0.547	0.298	0.302	0.433	0.509
RankBoost [16]	0.373	0.318	0.208	0.256	0.369	0.403
AdaRank [17]	0.288	0.362	0.173	0.228	0.196	0.391
CoordAscent [18]	0.466	0.681	0.241	0.293	0.494	0.539
LambdaRank [19]	0.156	0.155	0.235	0.213	0.429	0.495
LambdaMART [20]	0.646	0.749	0.279	0.392	0.515	0.569
ListNET [11]	0.433	0.626	0.191	0.240	0.470	0.464
RandForest [21]	**0.731**	**0.781**	0.383	0.366	0.298	0.431
MDPRank [6]	0.344	0.441	0.413	0.541	0.568	0.642
KFRank	0.425	0.444	**0.599**	**0.565**	**0.636**	**0.686**

Bold values represent the best performance of nDCG@k.

## Data Availability

The homework record data used to support the findings of this study were supplied by the Xuehai Education Technology Co., Ltd., in China. Since data would reveal personal activities and the size of data is huge, the data cannot be made freely available. We are glad to supply part of the data after removing the personal information and unique IDs for your research. Requests for access to these data and project code should be made to Canghong Jin with e-mail jinch@zucc.edu.cn.
